# Evaluation of the associations between changes in intraocular pressure and metabolic syndrome parameters: a retrospective cohort study in Japan

**DOI:** 10.1136/bmjopen-2015-010360

**Published:** 2016-03-24

**Authors:** Hiroshi Yokomichi, Kenji Kashiwagi, Kazuyoshi Kitamura, Yoshioki Yoda, Masahiro Tsuji, Mie Mochizuki, Miri Sato, Ryoji Shinohara, Sonoko Mizorogi, Kohta Suzuki, Zentaro Yamagata

**Affiliations:** 1 Department of Health Sciences, University of Yamanashi, Chuo City, Yamanashi, Japan; 2 Department of Ophthalmology, University of Yamanashi, Chuo City, Yamanashi, Japan; 3 Yamanashi Koseiren Health Care Center, Kofu City, Yamanashi, Japan; 4 Department of Pediatrics, University of Yamanashi, Chuo City, Yamanashi, Japan; 5 Center for Birth Cohort Studies, University of Yamanashi, Chuo City, Yamanashi, Japan

**Keywords:** Intraocular Pressure, Metabolic Syndrome X, Ageing, Diabetes Mellitus

## Abstract

**Objective:**

The contributions of highly correlated cardiovascular risk factors to intraocular pressure (IOP) are not clear due to underlying confounding problems. The present study aimed to determine which metabolic syndrome parameters contribute to elevating IOP and to what extent.

**Design:**

Retrospective cohort study.

**Setting:**

A private healthcare centre in Japan.

**Participants:**

Individuals who visited a private healthcare centre and underwent comprehensive medical check-ups between April 1999 and March 2009 were included (20 007 in the cross-sectional study and 15 747 in the longitudinal study).

**Primary and secondary outcome measures:**

Changes in IOP were evaluated in terms of ageing and changes in metabolic syndrome parameters. Pearson's correlation coefficients and mixed-effects models were used to examine the relationship of changes in IOP with ageing and changes in metabolic syndrome parameters in cross-sectional and longitudinal studies, respectively.

**Results:**

In the cross-sectional study, IOP was negatively correlated with age and positively correlated with waist circumference, high-density lipoprotein cholesterol (HDL-C) levels, triglyceride levels, systolic blood pressure (SBP), diastolic blood pressure (DBP) and fasting plasma glucose (FPG) levels. In the longitudinal multivariate analysis, the associated IOP changes were −0.12 (p<0.0001) mm Hg with male sex; −0.59 (p<0.0001) mm Hg with 10 years of ageing; +0.42 (p<0.0001) mm Hg with 1 mmol/L increase in HDL-C levels; +0.092 (p<0.0001) mm Hg with 1 mmol/L increase in triglyceride levels; +0.090 (p<0.0001) mm Hg with 10 mm Hg increase in SBP; +0.085 (p<0.0001) mm Hg with 10 mm Hg increase in DBP; and+0.091 (p<0.0001) mm Hg with 1 mmol/L increase in FPG levels.

**Conclusions:**

Elevation of IOP was related to longitudinal worsening of serum triglyceride levels, blood pressure and FPG and improvement in serum HDL-C levels.

**Strengths and limitations of this study**

- This study included a large sample size (20 007 participants in the cross-sectional analyses and 15 747 participants in the longitudinal analyses).
- The longitudinal multivariate analysis modelled lifestyle-related systemic parameters together that potentially affect intraocular pressure.
- The mixed-effects models enabled repeated measurements to quantify the change in intraocular pressure in relation to the change in lifestyle-related systemic parameters.
- Interventional research on effect of lifestyle modifications on intraocular pressure among ophthalmological patients is warranted.
- Intraocular pressure was measured by non-contact tonometry and not with a Goldmann applanation tonometer.

## Introduction

The Framingham Eye Study and the Baltimore Eye Survey revealed that 4–7% of people aged ≥40 years have elevated intraocular pressure (IOP).^1^ ^2^ Since the literature indicates that in patients with glaucoma, lower IOP within the normal range (10–21 mm Hg) decreases the risk of visual field deterioration in comparison with higher IOP within the normal range,^3–6^ evidence of IOP reduction is necessary.

Previous studies suggest possible moderate associations between IOP elevation and cardiovascular risk factors.^7^ ^8^ Physiology clearly explains systemic hypertension as a risk factor for IOP elevation^9^ ^10^; however, it remains unclear whether all cardiovascular risk factors are related to IOP elevation.^11–14^ For example, ageing, a well-known cardiovascular risk factor, may affect IOP, but the results of previous studies have not been consistent among Caucasians, Asians and African-Americans; ageing reportedly increases IOP among European and American populations^15–17^ but decreases it in Asians.^18–20^ Furthermore, even if several cardiovascular risk factors have deleterious effects on IOP, it is unclear to what extent the IOP elevation can be attributed to each risk factor.^21^ ^22^ Specifically, previous studies have focused on the fact that obesity and age-related factors are highly correlated and therefore result in confounding problems that make it difficult to estimate the contribution of each cardiovascular risk factor to IOP.^23–25^

Parameters indexed in the definition of metabolic syndrome are used to easily assess the lifestyles of healthy individuals and the related cardiovascular risks. Since the parameters are modified by changes in diet and physical activities, people are more likely to set their health goals at improving the parameters.^26^ Recent observational studies have suggested that lifestyle and physiological factors affect IOP in healthy individuals without glaucoma^27^ ^28^ and that along with blood pressure, other metabolic syndrome parameters such as waist circumference, plasma lipid levels and plasma glucose levels may also be associated with IOP elevation.^29^ ^30^ However, the analyses have not yet solved the aforementioned controversy regarding confounding problems of the correlated explanatory parameters. In addition, they have also not quantified the level of IOP increase associated with a deterioration in the metabolic syndrome parameters. This study aimed to determine which metabolic syndrome parameters elevate IOP and to what extent in a cohort of ophthalmologically healthy individuals who had undergone medical check-ups.

## Methods

### Study participants

This retrospective cohort study used data collected from residents of Yamanashi Prefecture, Japan, who visited a private healthcare centre and underwent a paid comprehensive medical check-up service between April 1999 and March 2009. To exclude the effect of ocular hypotensive therapy that would largely decrease IOP and affect the investigated associations, individuals with funduscopic findings during this period were excluded from the study. Only the first visit in a single fiscal year from April to March was considered for each participant. Hence, the maximum number of participant visits was 10 in the study data, and data from the second and subsequent visits during a fiscal year were excluded. For the cross-sectional study, we analysed data that were obtained between April 2008 and March 2009. For the longitudinal study, we included data for participants who had 3–10 visits between April 1999 and March 2009.

### Measurements

During the medical check-ups, we measured IOP, waist circumference, blood pressure and serum markers of metabolic syndrome. All medical measurements were recorded between 9:00 and 12:00. Serum markers were assessed from blood samples that were collected in the morning before breakfast. IOP was measured with a non-contact tonometer (NT-3000, Nidek, Tokyo), and IOP levels in the right eyes were analysed. Blood pressure was measured on the upper right arm while the participants were seated. The baseline body mass index (BMI) was calculated as weight in kilograms divided by the square of height in metres.

### Statistical analyses

To assess the relationship between metabolic syndrome and IOP in the cross-sectional study, Pearson's correlation coefficients with Bonferroni correction for multiple comparisons were calculated to determine the association between IOP and waist circumference, high-density lipoprotein cholesterol (HDL-C) levels, triglyceride levels, systolic blood pressure (SBP), diastolic blood pressure (DBP) and fasting plasma glucose (FPG) levels. To determine the association between IOP and the severity of metabolic syndrome in the cross-sectional study, the mean IOP levels were represented with respect to the numbers (0–5) of positive metabolic syndrome parameters according to the diagnostic criteria. The diagnostic criteria were based on the International Diabetes Federation guidelines.^31^ The five identified parameters of metabolic syndrome were a waist circumference of ≥85 cm in men and ≥90 cm in women; triglyceride levels of ≥1.7 mmol/L (150 mg/dL) or specific treatment for this lipid abnormality; HDL-C levels of <1.03 mmol/L (40 mg/dL) in men and <1.29 mmol/L (50 mg/dL) in women or specific treatment for this lipid abnormality; SBP of ≥130 mm Hg, DBP of ≥85 mm Hg or treatment of previously diagnosed hypertension; and FPG levels of >5.6 mmol/L (100 mg/dL) or previously diagnosed type 2 diabetes. The Jonckheere–Terpstra trend test was used to assess the significance of the trend in IOP with respect to the numbers of positive metabolic syndrome parameters.^32^ Univariate and multivariate mixed-effects models with a random intercept for participants were used to longitudinally assess the relationship between changes in IOP and metabolic syndrome parameters.^33^ Since waist circumference was measured only during the past three fiscal years (2006–2008) during the observed 10-year period, it was not included in the longitudinal analyses. Before conducting the multivariate analysis, we checked the multicollinearities among all the explanatory variables, particularly between SBP and DBP, which could seriously interfere with the estimates of interest.^34^ In terms of variance inflation factors ≤4, no multicollinearity was detected.^35^ The used model examples for the longitudinal analyses are represented below.
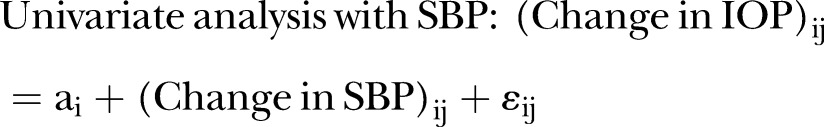

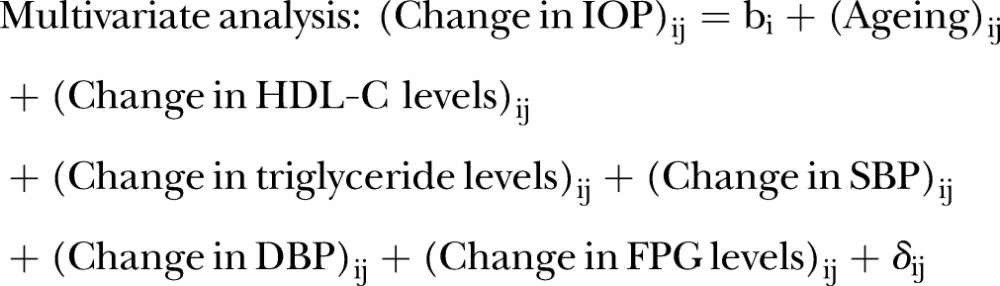
i for participants; j for time points.
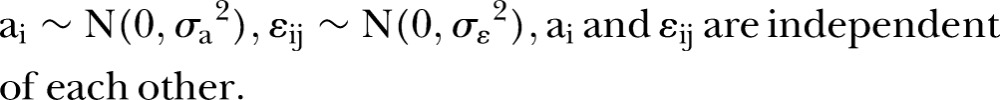

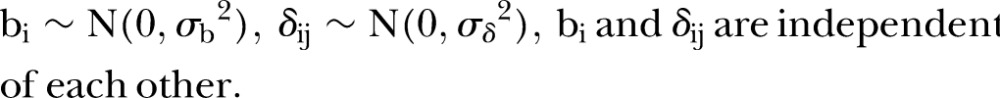


### Sensitivity analyses

To confirm the longitudinal results, we performed two sensitivity analyses: (1) The first sensitivity analysis was conducted using the data in which metabolic syndrome parameters and IOP could be measured one or more times in a single fiscal year. As a result, participants had 3–20 visits between April 1999 and March 2009. (2) The second sensitivity analysis was conducted using the data for which the age of participants was restricted to 19–44 years. This was done because the data suggested that participants aged less than 45 years were more likely to be lost to follow-up, and the main longitudinal results were deduced primarily from middle-aged participants.

All statistical analyses were performed using SAS statistical software (V.9.3, SAS Institute, Cary, North Carolina, USA) Descriptive statistics were reported as means and SDs; the point estimates were reported with 95% CIs. All reported p values were two-sided, and p<0.05 was considered statistically significant.

## Results

### Cross-sectional study

Table 1 shows the characteristics of the 10 122 men and 9885 women who were included in the cross-sectional analyses and underwent medical check-ups between April 2008 and March 2009. The mean (SD) values for men were as follows: age, 54.3 (11.6) years; IOP, 12.9 (2.9) mmg; BMI, 23.4 (2.9) kg/m^2^; waist circumference, 84.8 (7.9) cm; HDL-C levels, 1.39 (0.35) mmol/L or 53.9 (13.4) mg/dL; triglyceride levels, 1.46 (1.02) mmol/L or 129.1 (90.4) mg/dL; SBP, 121.3 (16.3) mm Hg; DBP, 77.1 (10.8) mm Hg; and FPG levels, 5.78 (1.07) mmol/L or 104.1 (19.3) mg/dL. The mean (SD) values for women were as follows: age, 54.8 (11.1) years; IOP, 12.5 (2.8) mm Hg; BMI, 22.0 (3.2) kg/m^2^; waist circumference, 79.4 (8.8) cm; HDL-C levels, 1.65 (0.36) mmol/L or 63.9 (14.0) mg/dL; triglyceride levels, 1.00 (0.56) mmol/L or 88.7 (49.9) mg/dL; SBP, 115.0 (17.2) mm Hg; DBP, 70.8 (10.7) mm Hg; and FPG levels, 5.37 (0.74) mmol/L or 96.6 (13.3) mg/dL. Table 2 represents Pearson's correlation coefficients for IOP in both sexes in relation to age and metabolic syndrome parameters. In men, IOP was negatively correlated with age levels and positively correlated with waist circumference, HDL-C levels, triglyceride levels, SBP, DBP and FPG levels. In women, IOP was positively correlated with waist circumference, triglyceride levels, SBP, DBP and FPG levels. Table 3 shows the means and SDs of the IOP levels stratified by the numbers of positive metabolic syndrome parameters. The analysed population in the cross-sectional study was restricted to 15 256 participants for whom data for all metabolic parameters, history and medication were available. Individuals with 0, 1, 2, 3, 4 and 5 positive metabolic syndrome parameters showed mean (SD) IOP of 12.2 (2.7), 12.6 (2.8), 12.9 (2.9), 13.3 (2.9), 13.3 (2.8) and 13.5 (2.8) mm Hg, respectively. The Jonckheere–Terpstra trend test detected statistical significance with p<0.0001 between the number of positive metabolic syndrome parameters and mean IOPs.

**Table 1:** Characteristics of participants who underwent a medical check-up between April 2008 and March 2009

Variables, mean (SD)	Men (n=10 122)	Women (n=9885)
Age, years	54.3 (11.6)	54.8 (11.1)
Intraocular pressure, mm Hg	12.9 (2.9)	12.5 (2.8)
Body mass index, kg/m^2^	23.4 (2.9)	22.0 (3.2)
Waist circumference, cm	84.8 (7.9)	79.4 (8.8)
HDL-C, mmol/L	1.39 (0.35)	1.65 (0.36)
(HDL-C, mg/dL)	53.9 (13.4)	63.9 (14.0)
Triglyceride, mmol/L	1.46 (1.02)	1.00 (0.56)
(Triglyceride, mg/dL)	129.1 (90.4)	88.7 (49.9)
Systolic blood pressure, mm Hg	121.3 (16.3)	115.0 (17.2)
Diastolic blood pressure, mm Hg	77.1 (10.8)	70.8 (10.7)
Fasting plasma glucose, mmol/L	5.78 (1.07)	5.37 (0.74)
(Fasting plasma glucose, mg/dL)	104.1 (19.3)	96.6 (13.3)

HDL-C, high-density lipoprotein cholesterol.

**Table 2:** Pearson's correlation coefficients of IOP with age and metabolic syndrome parameters in funduscopically healthy adults

	Pearson's correlation coefficient with IOP (95% CI)			
Parameters	Men (n=10 122)	p Value*	Women (n=9885)	p Value*
Age	−0.10 (−0.12 to −0.08)	<0.001	−0.003 (−0.02 to 0.02)	1.00
Waist circumference	+0.09 (0.08 to 0.11)	<0.001	+0.09 (0.07 to 0.11)	<0.001
HDL-C	+0.03 (0.006 to 0.05)	<0.01	+0.0002 (−0.02 to 0.02)	1.00
Triglyceride	+0.08 (0.06 to 0.10)	<0.001	+0.07 (0.05 to 0.09)	<0.001
Systolic blood pressure	+0.17 (0.16 to 0.19)	<0.001	+0.22 (0.20 to 0.24)	<0.001
Diastolic blood pressure	+0.17 (0.15 to 0.19)	<0.001	+0.19 (0.17 to 0.21)	<0.001
Fasting plasma glucose	+0.12 (0.10 to 0.14)	<0.001	+0.15 (0.14 to 0.17)	<0.001

*p Values for multiple comparisons were corrected by Bonferroni's method.

HDL-C, high-density lipoprotein cholesterol; IOP, intraocular pressure.

**Table 3:** Intraocular pressure (IOP) in relation to the numbers of positive metabolic syndrome parameters*

Number of positive metabolic syndrome parameters*	Number of participants	IOP, mm Hg, mean (SD)	p Value for linear trend
0	6171	12.2 (2.7)	<0.0001
1	4507	12.6 (2.8)	
2	2908	12.9 (2.9)	
3	1240	13.3 (2.9)	
4	363	13.3 (2.8)	
5	67	13.5 (2.8)	

*Five parameters were used for diagnosing a patient with metabolic syndrome: waist circumference of ≥85 cm in men and ≥90 cm in women; triglyceride levels of ≥1.7 mmol/L (150 mg/dL) or specific treatment for this lipid abnormality; high-density lipoprotein cholesterol levels of <1.03 mmol/L (40 mg/dL) in men and <1.29 mmol/L (50 mg/dL) in women or specific treatment for this lipid abnormality; systolic blood pressure of ≥130 mm  Hg, diastolic blood pressure of ≥85 mm  Hg or treatment of previously diagnosed hypertension and fasting plasma glucose levels of >5.6 mmol/L (100 mg/dL) or previously diagnosed type 2 diabetes in accordance with the definition of the metabolic syndrome in International Diabetes Federation.

### Longitudinal study

The longitudinal study included the data of changes in measured parameters in 15 747 participants. The mean (SD) number of visits during the 10-year period among the participants of the longitudinal analyses was 4.34 (2.53) for men and 4.03 (2.42) for women. The mean (SD) follow-up duration was 1711 (929) days for men and 1705 (881) days for women. Table 4 shows the estimated coefficients, 95% CIs and p values in the mixed-effects models, analysing the relationship between the change in IOP and the changes in the metabolic syndrome parameters. After adjusting for multiple cardiovascular risk factors, the change in IOP showed negative associations with male sex and ageing and positive associations with increases in HDL-C levels, triglyceride levels, SBP, DBP and FPG levels.

**Table 4:** Changes in intraocular pressure with ageing and changes in metabolic syndrome parameters: 10-year longitudinal study (n=15 747)

	Univariate analyses			Multivariate analysis		
Explanatory variable	Coefficient	95% CI	p Value	Coefficient	95% CI	p Value
Sex, men	−0.35	−0.39 to −0.32	<0.0001	−0.12	−0.16 to −0.08	<0.0001
Ageing, +10 years	−0.73	−0.77 to −0.69	<0.0001	−0.59	−0.64 to −0.54	<0.0001
HDL-C, +1 mmol/L	+0.28	0.21 to 0.35	<0.0001	+0.42	0.35 to 0.49	<0.0001
(HDL-C, +10 mg/dL)	+0.073	0.055 to 0.090	<0.0001	+0.11	0.09 to 0.13	<0.0001
Triglyceride, +1 mmol/L	+0.095	0.077 to 0.11	<0.0001	+0.092	0.073 to 0.11	<0.0001
(Triglyceride, +10 mg/dL)	+0.011	0.0086 to 0.013	<0.0001	+0.010	0.008 to 0.012	<0.0001
SBP, +10 mm Hg	+0.16	0.15 to 0.17	<0.0001	+0.090	0.077 to 0.10	<0.0001
DBP, +10 mm Hg	+0.20	0.19 to 0.22	<0.0001	+0.085	0.067 to 0.10	<0.0001
FPG, +1 mmol/L	+0.042	0.021 to 0.063	<0.0001	+0.091	0.071 to 0.11	<0.0001
(FPG, +10 mg/dL)	+0.023	0.012 to 0.035	<0.0001	+0.051	0.039 to 0.062	<0.0001

DBP, diastolic blood pressure; FPG, fasting plasma glucose; HDL-C, high-density lipoprotein cholesterol; SBP, systolic blood pressure.

### Sensitivity analyses

Table 5 shows the results of the two sensitivity analyses: (1) which allowed multiple measurements in a single fiscal year among the same 15 747 participants and (2) in which participant ages were restricted to 19–44 years (n=5261). In the first sensitivity analysis, the estimated coefficients for the longitudinal association between changes in metabolic syndrome parameters and IOP were almost similar to those of the original multivariate analysis. The second sensitivity analysis also suggested little differences in the estimated coefficients between the data of the younger adults and those of the total study participants.

**Table 5:** Multivariate analyses for the associations of changes in intraocular pressure with ageing and changes in metabolic syndrome parameters.

Explanatory variable	Sensitivity analysis (1), n=15 747			Sensitivity analysis (2), n=5261		
	Coefficient	95% CI	p Value	Coefficient	95% CI	p Value
Sex, men	−0.12	−0.16 to 0.08	<0.0001	−0.092	−0.16 to −0.02	0.01
Ageing, +10 years	−0.59	−0.64 to −0.55	<0.0001	−0.74	−0.83 to −0.64	<0.0001
HDL-C, +1 mmol/L	+0.42	0.35 to 0.49	<0.0001	+0.44	0.30 to 0.57	<0.0001
(HDL-C, +10 mg/dL)	+0.11	0.09 to 0.13	<0.0001	+0.11	0.08 to 0.15	<0.0001
Triglyceride, +1 mmol/L	+0.091	0.073 to 0.11	<0.0001	+0.096	0.065 to 0.13	<0.0001
(Triglyceride, +10 mg/dL)	+0.010	0.008 to 0.012	<0.0001	+0.011	0.007 to 0.014	<0.0001
SBP, +10 mm Hg	+0.091	0.079 to 0.10	<0.0001	+0.083	0.054 to 0.11	<0.0001
DBP, +10 mm Hg	+0.085	0.067 to 0.10	<0.0001	+0.073	0.036 to 0.11	<0.0001
FPG, +1 mmol/L	+0.091	0.070 to 0.11	<0.0001	+0.15	0.10 to 0.20	<0.0001
(FPG, +10 mg/dL)	+0.050	0.039 to 0.062	<0.0001	+0.085	0.058 to 0.11	<0.0001

(1) Residents underwent health check-ups 3–20 times during 10 years. (2) Residents aged 19–44 underwent health check-ups once per year during 10 years.

DBP, diastolic blood pressure; FPG, fasting plasma glucose; HDL-C, high-density lipoprotein cholesterol; SBP, systolic blood pressure.

## Discussion

### Main findings

The cross-sectional study indicated that an increase in age was a protective factor against elevated IOP, and increases in HDL-C, triglyceride, SBP, DBP and FPG levels were risk factors for elevated IOP. The longitudinal data over 10 years revealed that ageing decreased IOP and that worsening of triglyceride levels, SBP, DBP and FPG levels elevated IOP.

### Interpretation of the models

The cross-sectional and longitudinal analyses provided different interpretations of the association between metabolic syndrome and IOP. The cross-sectional study showed modest-to-moderate relationships at a single time point, independent of the units of metabolic syndrome parameters (table 2). The cross-sectional study also exhibited a dose–response relationship of the severity of metabolic syndrome to elevated IOP (table 3). Crude and confounder-adjusted changes in IOP per unit increase in each metabolic syndrome parameter as longitudinal associations are presented in table 4. We believe that this observational study answered the study question regarding which metabolic syndrome parameters contribute to changes in IOP as well as the magnitude of such changes.

### Results in the context of other studies

The results of this study agree with those of previous studies that described SBP^7^ ^36^ as a moderate risk factor for elevated IOP. Concerning ageing, the results have been inconsistent across populations; while results from Western populations have shown that ageing is positively correlated with IOP,^36^ ^37^ results from Asian populations have been consistent with ageing as a protective factor against high IOP.^18–20^ ^22^ ^23^ ^29^ ^38^ The present longitudinal results from a Japanese population favour this hypothesis about Asians. Ageing may have the potential to exert an ocular hypotensive effect after adjusting for confounders. In terms of glycated haemoglobin levels that reflect the month-to-month plasma glucose levels,^39^ other univariate longitudinal mixed-effects model analyses demonstrated that an increase of 10 mmol/mol in glycated haemoglobin levels is associated with a −0.030 mm Hg IOP change (95% CI −0.061 to 0.002, p=0.07) and that an increase of 1% in glycated haemoglobin levels is associated with a −0.032 mm Hg IOP change (95% CI −0.067 to 0.003, p=0.07). Although an observational study indicated diabetes mellitus as a risk factor for primary open-angle glaucoma,^12^ little is known about the association between high plasma glucose levels and IOP. This study, which showed a positive relationship with FPG levels and a negative relationship with glycated haemoglobin levels, did not provide a clear indication of the association between elevated plasma glucose levels and IOP. With respect to the effect of serum lipids on IOP, some previous studies have shown moderate positive correlations between serum triglyceride levels and IOP,^30^ ^40–42^ whereas another study found no association between these two factors.^43^ The present results were inconclusive about the association between HDL-C levels and IOP; our results demonstrated a small cross-sectional association and a moderate longitudinal association between HDL-C levels and IOP. Since elevated total cholesterol levels can be partly attributed to elevated HDL-C levels,^44^ and epidemiological studies have not yet revealed an association between IOP and serum lipids, physiological studies to investigate the presence or absence of this association would be necessary.

### Possible reasons for these associations

Studies have suggested possible mechanisms for the association between cardiovascular risk factors and elevated IOP. In particular, hypertension is linked to an elevated IOP in a physiological manner; SBP, rather than DBP, elevates IOP because peaks of SBP that reach the eye can lead to ultrafiltration.^45–47^ On the other hand, although epidemiological studies have reported obesity as a risk factor for increased IOP,^48^ it has not been clearly explained how lifestyle-related factors elevate IOP. Although unproven, one study attributed this phenomenon observed in obese individuals to the fact that obesity produced excess intraorbital fat tissue, increased episcleral venous pressure and blood viscosity.^49^ Another study in a Korean population, using insulin resistance as an index mediating all obesity-related systemic factors, indicated the necessity of further cohort studies that handle respective obesity-related parameters as independent exposure variables.^50^ With respect to the high prevalence of elevated IOP that has been observed among patients with diabetes,^36^ ^48^ diabetes-related autonomic dysfunction, genetic factors and corneal stiffening may partly explain elevated IOP.^24^ The mechanisms of ageing, which is a risk factor for elevated IOP in Westerners but not in Asians, may be explained by the high prevalence of obesity, hypertension and diabetes in aged Westerners.^51^ ^52^ It has been speculated that the ocular hypertensive effects of ageing in Westerners may be a result of the ocular hypertensive effects of obesity, hypertension and diabetes that surpass the actual hypotensive effects of ageing. Concerning the moderate positive association between serum triglyceride levels and IOP in this study, there has been no comparable literature. Since statins reportedly decrease the risk of open-angle glaucoma,^53^ individuals who had been treated with statins and had relatively low IOP in the data may have produced the observed moderate association between IOP elevation and high serum triglycerides. Whereas age,^16^ African-Americans,^54^ ^55^ family history,^56^ hypertension,^9^ diabetes^57^ and elevated IOP^58^ are known risk factors for open-angle glaucoma, the previous and present studies have suggested that age is a protective factor in Asians, and hypertension^9^ ^10^ and high plasma glucose^36^ ^48^ are risk factors for elevated IOP.

### Strengths and limitations

The following three factors were the strengths of this study. First, we believe that modelling IOP in relation to ageing, serum lipids, blood pressure and plasma glucose together estimated the amount of IOP attributable to lifestyle-related systemic parameters and addressed the underlying confounding problems. As shown in table 3, the increase in the severity of the metabolic syndrome resulted in a linear increase in IOP. We consider that the consistency between the cross-sectional and longitudinal results reflects the internal validities. Second, the number of participants and the follow-up period in this study were sufficient to investigate the study question. We propose that the results of the study could be extrapolated to Japanese populations. Third, the mixed-effects models in table 4 incorporated repeated measurements and could detect the longitudinal change-to-change relationships.

This study has some limitations. The first is that the data included ophthalmologically healthy participants, most of whom were middle-aged or older. Although the results could be applied to ophthalmologically premedicated people, a study about the intervention of lifestyle modifications on IOP among ophthalmological patients is warranted to ensure applicability to all ophthalmological patients. In addition, since the main longitudinal regression results were deduced primarily from middle-aged individuals, the associations may not be directly applied to younger generations. However, the sensitivity analysis (2) confirmed that the associations in younger generations and those in the total population were almost similar. Another limitation is that IOP was measured using non-contact tonometry. It has been reported that IOP measured by this device is not directly comparable to IOP measured by the Goldmann applanation tonometer, the gold standard instrument. Since the difference in measurements between the Goldmann applanation tonometer and non-contact tonometry may increase with ageing,^59^ IOP measurements would have been more accurate if the thicknesses of the corneas had been measured simultaneously and used to adjust the IOP measurements. This means that IOP in the present data may have been negatively biased in middle-aged or older participants. However, we believe that the ability of the mixed-effects model to estimate the change-to-change relationships minimised this systematic bias. Lastly, this study did not include actual lifestyle-related variables, such as diet, physical activities, smoking, other diseases and socioeconomic status. The study suggests that if people improve their metabolic syndrome parameters by modifying their lifestyles, their IOP will decrease; however, a study with lifestyle interventions would be necessary to confirm this suggestion.

## Conclusion

The longitudinal studies revealed that deterioration of waist circumference, blood pressure and FPG accompany the elevation of IOP. The results also suggest that an increase in HDL-C levels accompanies an elevation of IOP; therefore, the results should be carefully interpreted, and further physiological investigations regarding serum lipids and IOP are necessary.
